# Weakly Hard Real-Time Model for Control Systems: A Survey

**DOI:** 10.3390/s23104652

**Published:** 2023-05-11

**Authors:** Karla Salamun, Ivan Pavić, Hrvoje Džapo, Ivana Čuljak

**Affiliations:** Faculty of Electrical Engineering and Computing, University of Zagreb, Unska 3, 10000 Zagreb, Croatia; ivan.pavic2@fer.hr (I.P.);

**Keywords:** weakly hard real-time systems, real-time task scheduling, control and scheduling co-design

## Abstract

The concept of weakly hard real-time systems can be used to model real-time systems that may tolerate occasional deadline misses in a bounded and predictable manner. This model applies to many practical applications and is particularly interesting in the context of real-time control systems. In practice, applying hard real-time constraints may be too rigid since a certain amount of deadline misses is acceptable in some applications. In order to maintain system stability, limitations on the amount and distribution of violated deadlines need to be imposed. These limitations can be formally expressed as weakly hard real-time constraints. Current research in the field of weakly hard real-time task scheduling is focused on designing scheduling algorithms that guarantee the fulfillment of constraints, while aiming to maximize the total number of timely completed task instances. This paper provides an extensive literature review of the work related to the weakly hard real-time system model and its link to the field of control systems design. The weakly hard real-time system model and the corresponding scheduling problem are described. Furthermore, an overview of system models derived from the generalized weakly hard real-time system model is provided, with an emphasis on models that apply to real-time control systems. The state-of-the-art algorithms for scheduling tasks with weakly hard real-time constraints are described and compared. Finally, an overview of controller design methods that rely on the weakly hard real-time model is given.

## 1. Introduction

Control systems play a crucial role in modern engineering, enabling the precise and efficient regulation of complex physical processes. They are widely used in diverse applications to ensure stability, accuracy, and safety. However, designing effective control systems is a challenging task that requires a deep understanding of the system dynamics, modeling techniques, and control strategies.

Conventional approaches in control systems design utilize conservative principles in terms of temporal constraints. The execution of control tasks is bounded to fixed time slots that are determined according to the worst-case execution time of the tasks. Moreover, jitter in the task activation time is not allowed. Such constraints can be classified as hard real-time constraints, where missing a task deadline is strictly forbidden as it may cause catastrophic damage to the system and its environment. Moreover, the control tasks are typically scheduled according to fixed-priority policies in order to guarantee predictability.

The research in real-time scheduling theory is focused on dimensioning the real-time system such that a given resource is maximally utilized, and the methods for reducing the system load often rely on the relaxation of hard real-time constraints. Furthermore, the advanced scheduling techniques rely on dynamic priority assignment since, in the general case, it is not possible to fully utilize the CPU (Central Processing Unit) under static scheduling schemes [1]. Although established methods for control system’s design implement some basic principles of real-time scheduling, the separate development of these two research areas has led to a gap between the problem domains in control and real-time scheduling. As a consequence, the results and design methods from the real-time scheduling theory are hardly applicable to techniques for control systems design [2]. Integrating more advanced scheduling techniques into control systems can be highly beneficial since it allows for a higher degree of flexibility in the system design and the possibility of designing more cost-effective control systems. The main challenge is to achieve a trade-off between control performance and efficient computing resource utilization. Therefore, integrating the control and real-time scheduling research field has become a great challenge, both in the industrial and academic communities.

Most of the methods for control and scheduling co-design known from the literature rely on the relaxation of hard real-time constraints. Relaxing the hard real-time constraints usually implies that violating the task deadline is allowed under certain conditions. For many practical applications, including real-time control systems, the hard real-time constraints can be too rigid because the system can tolerate occasional deadline misses if the amount and distribution of missed deadlines follow a specific pattern. Due to the robust design of real-time control systems, occasional deadline misses that are adequately constrained have negligible consequences on system performance; they can cause quality of service degradation, but they do not jeopardize the system stability [3,4]. Moreover, the ability to miss a certain degree of deadlines provides more scheduling slack and flexibility and thus permits the design of more cost-effective systems.

On the other hand, in soft real-time systems, deadline misses are tolerable since they still bring some utility to the system. However, the soft real-time system model does not provide any mechanisms for constraining the ratio and consecutiveness of violated deadlines. The weakly hard real-time system model is introduced to bridge the gap between hard and soft real-time systems, and it provides a framework for describing the characteristics of a system that can tolerate a certain degree of deadline misses under a precise distribution. In order to define the temporal constraints for specifying the degree and distribution of tolerable deadline misses, the concept of weakly hard real-time constraints is introduced [4].

The first application of the weakly hard real-time model was in the context of scheduling messages in communication systems [5]. The initial model was further extended to the problem of scheduling tasks on a single CPU [6,7] and multiprocessor systems [8]. In the literature, there are many applications that rely on the weakly hard model for defining the temporal constraints, including networked systems [9,10], control systems [11,12,13], cyber-physical systems [14,15,16], communication systems [17,18], and multimedia systems [19,20,21]. In recent years, research directions in the area of weakly hard real-time systems include schedulability analysis [22,23,24,25], energy-constrained scheduling [26,27,28,29], control systems stability analysis [30,31,32], and control and scheduling co-design [33,34].

There are a plethora of recent survey papers that cover novel methods for control systems’ design [35,36,37,38] and only a few papers provide a review of the scheduling approaches based on the weakly hard real-time system model [39,40]. In the context of scheduling and control systems co-design, there are several existing surveys that cover this topic. However, they mostly consider networked control systems [41,42], and from the scheduling aspect, they deal with the analysis of overrun handling strategies rather than the analysis of different scheduling algorithms [43]. In this paper, we aim to consolidate recent research from the field of scheduling and control co-design based on the weakly hard real-time system model and systematize the existing scheduling algorithms for weakly hard real-time systems, which are not covered in any survey papers so far. The goal of this work is to provide prospective researchers of the weakly hard real-time systems field with an extensive background of the existing system models, scheduling algorithms, and schedulability analysis approaches. Moreover, a link to the research field of control systems design is explained, as well as the most important results in the area of scheduling and control systems’ co-design. A literature review of the following research topics is provided:

- The weakly hard real-time models presented in the literature and their applications;
- The existing approaches for scheduling tasks under weakly hard real-time constraints;
- The existing methods for analyzing the schedulability of weakly hard real-time systems;
- Recent work on control and scheduling co-design, focusing on the approaches that rely on the weakly hard real-time model.

The rest of the paper is organized as follows. Section 2 describes the task model from classical real-time scheduling theory and the motivation for using the weakly hard real-time model. The system models known from the literature, which can be derived from the generalized weakly hard real-time model, are described in Section 3. Section 4 provides an overview of the algorithms for scheduling task sets with weakly hard constraints, while the approaches for performing a feasibility analysis of such task sets are described in Section 5. The challenges and recent research in the field of control and scheduling co-design are described in Section 6. Section 7 gives a conclusion and outlines the open challenges for future research.

## 2. Preliminaries

### 2.1. System Model

In the general real-time system model, a real-time system is represented by a set of *N* tasks: $T=\{\tau_{1},\tau_{2},\ldots ,\tau_{N}\}$, where each task is described by means of three temporal parameters: period $T_{i}$, deadline $D_{i}$, and worst-case execution time $C_{i}$. Each task $\tau_{i}$ releases an infinite sequence of instances, i.e., jobs. The *j*-th job of a task $\tau_{i}$ is denoted as $J_{ij}$. For periodic tasks, the separation between two consecutive jobs is exactly one period $T_{i}$. A hyperperiod H is the smallest interval of time after which the schedule of periodic tasks is repeated. Generally, the hyperperiod corresponds to the least common multiple of the task periods: H = lcm $\left(T_{1},T_{2},\ldots ,T_{N}\right)$. If the first job of a task is not released at time t=0, the elapsed time before the first release of the job is called the offset $O_{i}$ of the task $\tau_{i}$. In the general case, the invocation time of the *j*-th job of task $\tau_{i}$, denoted by $S_{ij}$, equals $S_{ij}=\left(j-1\right)T_{i}+O_{i}$. The deadline $D_{i}$ corresponds to the time by which a job of task $\tau_{i}$ must complete its execution. The deadline specified with respect to the job arrival time is called a relative deadline, whereas a deadline specified with respect to time t=0 is an absolute deadline. A specific case where the relative deadline is equal to the task period, i.e., $D_{i}=T_{i}$, is referred to as an implicit deadline. The task deadline is the most widely used parameter for defining the temporal constraints of the system and defining the feasibility conditions for scheduling algorithms. If a job has completed its execution before the deadline, it is said that the job has met the deadline, otherwise, the deadline is missed. A schedule is considered feasible if all jobs meet their deadlines. The response time for job $J_{ij}$ is denoted by $R_{ij}$ and it is computed as $R_{ij}=F_{ij}-S_{ij}$, where $F_{ij}$ is the finalization time of job $J_{ij}$. The worst-case response time of a task $\tau_{i}$ is the maximum response time among all of the jobs of task $\tau_{i}$: $R_{i}=\max_{j}\left\{R_{ij}\right\}$. A task $\tau_{i}$ is deemed schedulable if and only if $R_{i}\leq D_{i}$ [44].

The utilization factor *U* for a given set T of *N* periodic tasks is defined as a fraction of the processor time spent in the execution of the task set T [1]:

(1) $$U=\sum_{i=1}^{N}\frac{C_{i}}{T_{i}}$$

If U>1, then no feasible schedule exists for the task set.

One of the most important concepts in the schedulability analysis is the critical instant, i.e., the arrival time of a job that produces the maximum response time among all of the jobs of task $\tau_{i}$. The schedulability tests typically rely on the critical instant theorem [1], which states that a critical instant for a task $\tau_{i}$ occurs whenever a job of task $\tau_{i}$ occurs simultaneously with the higher-priority jobs [45,46].

Weakly hard real-time systems can be characterized by extending the described system model with weakly hard temporal constraints. The weakly hard constraints are used for specifying the degree and distribution of tolerable deadline misses. If the constraints are violated, the system is said to be in a dynamic failure [5].

**Definition** **1** (Dynamic failure). *A stream with (m,k)-firm deadlines experiences a dynamic failure if fewer than m customers meet their deadlines in a window of k consecutive customers.*

There are several process models known from the literature that specify these constraints:

- (m,k)-firm model,
- Skip-over model,
- Generalized weakly hard real-time model.

The (m,k)-firm model is introduced in the context of scheduling messages in communication systems [5]. The (m,k)-firm constraints specify that at least *m* deadlines must be met in any *k* consecutive invocations. The skip-over model is an approach that covers a special case where no consecutive deadline misses are allowed [47]. This model uses the notation of skip factor $s_{i}$, which specifies that only a single deadline can be missed in a window of $s_{i}$ consecutive invocations of a task $\tau_{i}$. The applications of the skip-over model include real-time control systems and multimedia systems. In real-time control systems, consecutive deadline misses would jeopardize the stability of the system, whereas in multimedia systems, consecutive deadline misses would degrade the quality of service. The generalized weakly hard real-time system model provides a common set of constraints that describe both the (m,k)-firm model and the skip-over model. Moreover, they describe constraints on the amount and consecutiveness of missed deadlines that are not covered by the (m,k)-firm and skip-over model. The (m,k)-firm, skip-over, and generalized weakly hard model are described in detail in Section 3.

It is important to emphasize that depending on the application, the system models differ with respect to the strategy for handling missed deadlines. The most common approaches are the *job-kill* and *job-continue* strategy [48]. In the job-kill method, the computation of a non-completed job is discarded at the deadline instant and the job is aborted. This approach applies to the skip-over model. With the job-continue policy, the jobs are always executed until completion and this approach is typically used with the (m,k)-firm model.

### 2.2. Motivation and Industrial Applications

This section brings a practical example of a real-time control system that illustrates the motivation for analyzing such systems through the weakly hard real-time model. It is demonstrated that designing the system according to the weakly hard real-time model can ensure predictive behavior in overload conditions. Moreover, weakly hard constraints can provide a convenient mechanism for resolving overload conditions.

**Figure 1:**
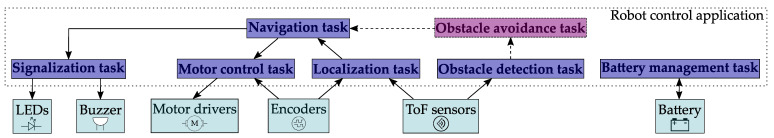
Example of an autonomous mobile robot (AMR) control system.

**Example** **1** (Computer-driven control system). *Consider a real-time system used for controlling an autonomous mobile robot (AMR). The AMR is battery-powered and equipped with time-of-flight (ToF) sensors used for localization and obstacle detection. Localization also relies on the encoders that measure the rotational speed of the wheels. The AMR is equipped with light-emitting diodes (LEDs) and buzzers for visual and audio signalization. Each functionality of the control system is carried out by a periodic task: localization, navigation, obstacle detection, battery management, motor control, and signalization. An additional task, i.e., obstacle avoidance task, is activated upon obstacle detection and it stays active until a trajectory that avoids the obstacle is computed. This task also releases periodic instances. A schematic view of the tasks and hardware components (sensors, actuators, etc.) is shown in Figure 1. The timing characteristics of the tasks are summarized in Table 1.*

*The system is fully utilized in normal operating conditions, i.e., when obstacle avoidance is inactive:*

(2) $$U_{nominal}=\sum_{i=1}^{N}\frac{C_{i}}{T_{i}}\frac{2}{10}+\frac{3}{10}+\frac{1}{10}+\frac{2}{20}+\frac{2}{10}+\frac{1}{10}=1$$

*Upon the activation of the obstacle avoidance task $\tau_{7}$, the total utilization factor becomes:*

(3) $$U_{max}=U_{nominal}+\frac{1}{10}=1.1$$

*and the system is temporarily under overload. In this example, overload is resolved by applying the weakly hard real-time constraints.*

*In normal operating conditions, the signalization task is intended to be invoked with the same frequency as the navigation task in order to properly indicate the motion of the AMR to the humans in its nearby environment, e.g., changing the LED light color when AMR changes direction, turning on the buzzer signal when AMR is reversing, etc. However, during system design, the signalization task was assigned the constraint (1, 5), which means that at least one job in five consecutively released jobs must be completed–in other words, four out of five jobs may be discarded if necessary. This means that, in the worst case, every fifth output of the navigation task will be signalized. Even in the worst-case scenario, the functionality of this task will not be affected by discarding jobs since refreshing the LEDs with a frequency of 20 Hz instead of 100 Hz is not noticeable by the human eye. Moreover, in the worst case, the buzzer will be triggered 50 ms later than the corresponding navigation action occurs and this delay is also tolerable.*

*The battery management task is responsible for monitoring the state of the battery, estimating the power consumption, and assessing when to schedule charging. This task is assigned a weakly hard constraint through a skip factor $s_{i}=5$–after one discarded job, four jobs in a row must be completed. This is possible since the inputs of this task are not changed frequently, and the robust design of the battery management algorithm allows for the stable operation of the battery management system (BMS) even in the worst-case scenario when every fifth job is discarded.*

*The scheduling algorithm is designed to schedule only mandatory jobs in overload conditions. If we take into account the maximum amount of skippable instances of the signalization and battery management tasks, the total utilization factor becomes:*

(4) $$U_{reduced}=\frac{2}{10}+\frac{3}{10}+\frac{1}{10}+\frac{4}{5}\cdot \frac{2}{20}+\frac{2}{10}+\frac{1}{5}\cdot \frac{1}{10}+\frac{1}{10}=1$$

*Overload is successfully resolved without jeopardizing the stability and degrading the performance of the system. Once the operation of the obstacle avoidance task is completed, the system returns to normal operating mode.*

*It is important to emphasize that the assumptions regarding the constraints on battery management task could also be made for other tasks (motor control, navigation, localization); however, for simplicity, the skip-over model was applied to only a single task.*

This example shows how weakly hard constraints can not only ensure deterministic behavior in overload conditions, but also provide a mechanism for designing a cost-effective system that is maximally efficient in normal conditions, while in the overload conditions the per-task utilization factors can be decreased in a predictable manner. Modeling the considered real-time system with hard real-time constraints requires dimensioning the system in a way so that in the worst case all deadlines are met. The demand of the tasks in the worst case compared to normal operation is typically even higher than shown in this example, and if the system is dimensioned according to the worst-case demand, it cannot be fully utilized in the normal operation mode. Thus, relaxing the hard temporal constraints may be beneficial in terms of a better utilization of the resources. On the other hand, applying soft real-time constraints to tasks that allow relaxed temporal constraints (signalization and battery management task) is not adequate as the pattern of missed deadlines cannot be guaranteed and missing a significant number of consecutive deadlines can harm the stability of the system and degrade performance. Moreover, the common techniques for scheduling systems under soft temporal constraints [49,50,51] are based on reducing the response time of the tasks without taking into account the per-task ratio of deadline violations. Therefore, for specific applications such as the control system shown in the example, it is reasonable to consider temporal constraints that are less stringent than the hard real-time requirements, but more stringent than soft real-time requirements in order to design cost-effective systems with precisely constrained temporal behavior.

## 3. Overview of Weakly Hard Real-Time System Models

### 3.1. Task Model with (m,k)-Firm Deadlines

**Figure 2:**
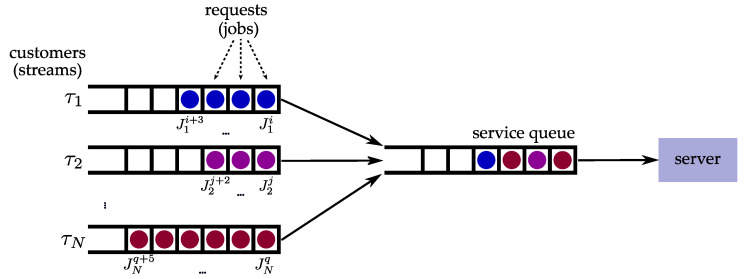
System model with (m,k)-firm constraints. Modified from [5]. The streams are denoted as $\tau_{i}$, while the *j*-th request of the *i*-th stream is denoted as $J_{i}^{j}$.

The earliest research related to systems that can tolerate the occasional loss of deadlines was the work of Hamdaoui and Ramanathan. In [5], they introduced the notion of (m,k)-firm deadlines as a specification of temporal constraints in the context of scheduling messages in communication systems. More specifically, their system model consists of streams of schedulable entities, which they refer to as customers, that must be scheduled on a single server. This system model can be used to represent many computer and telecommunication systems, including multiple tasks’ execution on a CPU and the transmission of messages from multiple streams that share the same medium. Each customer has a specified deadline $D_{i}$ by which the full service of the system is expected. If the customer is not fully serviced by the deadline, it is considered that the deadline is missed. The time required for serving the customer is referred to as the service time and it is equivalent to $C_{i}$. The service times for all streams are identical and service cannot be preempted. The minimum inter-arrival time between the requests of the customers is equivalent to the period $T_{i}$. Therefore, each stream can be described as a task $\tau_{i}\left(C_{i},D_{i},T_{i}\right)$, while the customer request corresponding to a stream is equivalent to a job $J_{ij}$. For simplicity, the terms *task* and *job* are used hereafter to represent streams and the corresponding customers. Each task is assigned two parameters, *m* and *k*, which specify that at least *m* jobs must meet their deadlines in any window of *k* consecutive requests of the task. If the stated requirement is not fulfilled, the task has experienced a dynamic failure. It is worth noting that this demand implies the maximum deadline miss rate of (k−m)/k. The described system model is depicted in Figure 2.

The problem addressed in the research of real-time systems with (m,k)-firm deadlines is scheduling the jobs of each task to a single server in such a way that dynamic failure is avoided.

### 3.2. Skip-Over Model

The skip-over model was introduced by Koren and Shasha in [47]. The authors address the problem of scheduling preemptable, periodic tasks that allow occasional deadline misses. The task deadlines are considered implicit, i.e., equal to the task periods. This model employs the job-kill strategy and jobs that miss their deadline are considered as skipped. The behavior of the system is analyzed in overload conditions and the constraints of job-skipping are fairly conservative; it is not allowed for two consecutive jobs to miss their deadlines.

The classical task model is extended by introducing a skip factor $s_{i}$, which specifies the tolerance of task $\tau_{i}$ to skipped jobs. More specifically, the skip factor defines the minimum requirement for the number of jobs that must meet their deadlines between two jobs that missed their deadlines. If a job of task $\tau_{i}$ is skipped, at least $\left(s_{i}-1\right)$ subsequent jobs must meet their deadlines. It is worth noting that $s_{i}=\infty$ corresponds to a hard real-time system where no deadline skips are allowed. For classifying whether a job is skippable, the authors propose a terminology of red and blue jobs. A red job must complete before its deadline, while a blue job may miss its deadline.

### 3.3. Generalized Weakly Hard Real-Time System Model

A weakly hard real-time system can be formally defined as [4]:

**Definition** **2** (Weakly hard real-time system). *A weakly hard real-time system is a system for which the distribution of its met and missed deadlines during a time window w is precisely bounded.*

In order to describe the requirements of a weakly hard real-time system, the weakly hard real-time model was introduced in [4]. The classical periodic task model is extended by a weakly hard constraint λ, which specifies the requirements on the pattern of met or missed deadlines: $\tau_{i}=\left(T_{i},D_{i},C_{i},\lambda_{i}\right)$. The temporal constraints λ are summarized in Table 2 and formally specified in the following definitions [52].

**Definition** **3** (Constraint nm). *A task “meets any n in m” deadlines (m≥1, 0≤n≤m) and is denoted by nm if in any window of m consecutive invocations of the task there are at least n invocations in any order that meet the deadline.*

**Definition** **4** (Constraint nm). *A task “meets row n in m” deadlines (m≥1, 0≤n≤m) and is denoted by nm if in any window of m consecutive invocations of the task there are at least n consecutive invocations that meet the deadline.*

**Definition** **5** (Constraint nm¯). *A task “misses any n in m” deadlines (m≥1, 0≤n≤m) and is denoted by nm¯ if in any window of m consecutive invocations of the task no more than n deadlines are missed.*

**Definition** **6** (Constraint nm¯). *A task “misses row n in m” deadlines (m≥1, 0≤n≤m) and is denoted by nm¯ if in any window of m consecutive invocations of the task there are no n consecutive invocations that miss the deadline.*

For the nm¯ constraint, the parameter *m* is not required because the satisfaction of the constraint does not depend on the size of the window; therefore, nm¯≡〈n〉¯.

It is worth noting that the constraints introduced by previous researchers can be directly modeled by the described constraints:

- The (m,k)-firm constraints from [5] can be directly mapped to the mk constraint;
- The constraints defined through skip factor $s_{i}$ from [47] correspond to si−1si.

For simplicity, in the rest of the paper the (m,k)-firm constraints are denoted as nm constraints and the constraints that correspond to the skip-over model are denoted as m−1m.

The history of met and missed deadlines for each task is often described through a binary sequence called the μ-pattern. The μ-pattern contains sufficient information needed for verifying that a task is satisfying its weakly hard constraints.

**Definition** **7** (μ-pattern). *A μ-pattern $\alpha_{i}$ of a task $\tau_{i}$ is a sequence of symbols {0,1}, where 1 denotes a met deadline and 0 denotes a missed deadline of the j-th job. The j-th symbol of $\alpha_{i}$ is computed in the following way:*

(5) $$\alpha_{i}\left(j\right)=\left\{\begin{array}{cc} 1 & \mathrm{if}\;R_{ij}\leq D_{i}\\ 0 & \mathrm{otherwise} \end{array}\right.$$

The length of the μ-pattern corresponds to the number of deadlines under a weakly hard constraint. An example of a μ-pattern that satisfies the constraint “4 out of 5 deadlines must be met” is 11011. The leftmost bit corresponds to the deadline of the oldest invocation. If the following invocation misses the deadline, the μ-pattern becomes 10110 and the corresponding task is in a failure state.

Weakly hard constraints are used in two different contexts: feasibility analysis and the implementation of new scheduling algorithms. In the context of feasibility analysis, the goal is to determine whether a task set scheduled by a given algorithm satisfies the weakly hard constraints. In the implementation of scheduling algorithms, μ-patterns are used for performing online computations of the probability of dynamic failure.

### 3.4. Research Problems

Considering the described specifications of weakly hard real-time systems, there are several problems that should be addressed:

1. Specifying the weakly hard temporal constraints;
2. Analyzing the schedulability of the system under weakly hard constraints;
3. Implementation of the algorithms for scheduling systems under weakly hard constraints.

The problem of specifying the temporal constraints for weakly hard real-time systems is thoroughly elaborated in [52]. The problem of analyzing the schedulability for tasks under weakly hard constraints cannot be addressed by applying the existing methods for analyzing hard real-time systems since the critical instant theorem does not hold in the case of weakly hard real-time systems [48]. The problem of scheduling systems with weakly hard constraints comes down to utilizing the weakly hard constraints to decrease the system load and, therefore, to be able to schedule a system that would otherwise be infeasible. The existing scheduling approaches are usually classified as guaranteed or best-effort. For guaranteed scheduling algorithms, there exists a schedulability test that guarantees that no dynamic failure can occur, whereas non-guaranteed schedulers use best-effort techniques to avoid dynamic failure. Best-effort approaches are often compared with respect to the probability of dynamic failure. The probability of dynamic failure is a measure of how often the weakly hard constraints are violated. Another metric used for comparing the performance of scheduling algorithms is the quality of service (QoS). QoS represents the ratio of completed jobs with respect to the total number of jobs:

(6) $$\mathrm{QoS}=\frac{\mathrm{number}\;\mathrm{of}\;\mathrm{completed}\;\mathrm{jobs}}{\mathrm{total}\;\mathrm{number}\;\mathrm{of}\;\mathrm{jobs}}$$

Guaranteed approaches typically employ offline priority assignment, where a pre-built sequence that determines which jobs will be skipped is constructed. Online approaches make scheduling decisions at runtime, usually based on dynamic parameters of the system. The complexity of the scheduling approach usually determines whether the approach is adequate for a certain application. For instance, computationally expensive schedulability tests are not adequate for online applications.

## 4. Overview of Scheduling Approaches

### 4.1. Scheduling Approaches for (m,k)-Firm System Model

Hamdaoui and Ramanathan have introduced distance-based priority assignment (DBP) as an approach for reducing the average probability of dynamic failure [5]. In this technique, the state of each task is tracked during the runtime in order to identify the tasks that are close to the failing state, i.e., state where fewer than *m* deadlines are met. The probability of dynamic failure is reduced by assigning the highest priorities to the jobs of tasks that are closest to the failing state. Each job is assigned a priority value equal to the minimum number of consecutive misses required to take the task from the current state to the failing state. In this case, the higher priority is given to customers with lower priority values.

**Figure 3:**
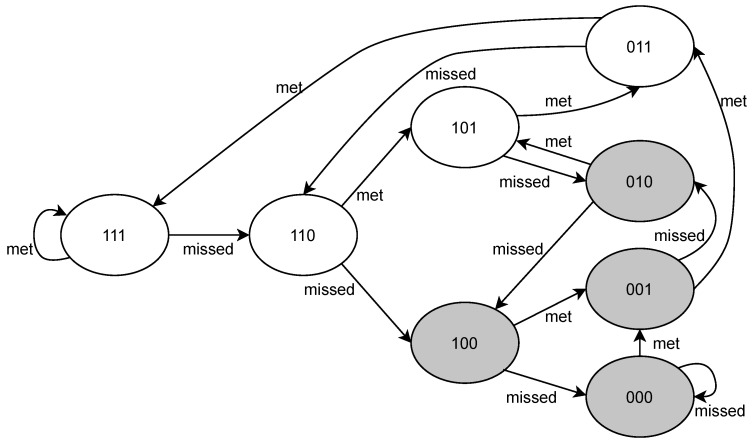
Transition diagram for a task with 23 constraint. Modified from [5].

The proposed priority assignment technique can be illustrated by a state transition diagram. For example, consider a task $\tau_{i}$ with 23 constraint. Figure 3 shows the state transition diagram for task $\tau_{i}$. The initial μ-pattern is 111. The edges of the diagram represent the possible state transitions (deadline missed or deadline met). If a state does not satisfy the nm constraint, the task is in the failing state, which is denoted by a shaded shape. The task in the failing state is assigned a priority value of 0, i.e., the highest priority, while the other tasks are assigned a priority corresponding to the distance to the failing state. For example, a task with the μ-pattern 110 is assigned priority 1, while a task with the μ-pattern 111 is assigned priority 2.

In comparison with scheduling schemes that use the Earliest Deadline First (EDF) algorithm, this approach brings a significant reduction in the probability of dynamic failure. However, a possible drawback of the DBP approach is that it assigns a priority only considering the current task, without considering the μ-patterns of the other tasks that share the same server. Moreover, the DBP priority assignment is based only on the history of missed and met deadlines of the tasks, not taking into account other properties such as deadlines, minimum inter-arrival time, etc.

In their further work, Hamdaoui and Ramanathan presented an analytic model for computing the probability of a dynamic failure [53]. This method can be used for providing statistical quality of service guarantees to real-time tasks.

The DBP approach was further investigated in the context of multi-hop network scheduling in [54], where an extended scheduling policy DBP-M was introduced. In this approach, the priorities of the tasks are adjusted dynamically in order to reduce the number of consecutive deadline misses and thus reduce the probability of dynamic failure. The priority for each task is computed according to the state information, which is maintained in a separate queue for each task. It is worth noting that the number of queues increases with the number of tasks and, therefore, this method incurs overhead in terms of computational and memory requirements. However, this approach outperforms classical DBP with respect to the probability of dynamic failure.

In [55], the problem of scalability in the existing best-effort approaches for scheduling systems with nm constraints is addressed. In this context, scalability refers to the property of the scheduling algorithm to handle an increase in the number of tasks without introducing a high cost in terms of the computational requirements. The authors point to the trade-off between the performance considering dynamic failures and the scheduling cost involved in achieving that performance. DBP-M requires a significant amount of scheduling cost to maintain a separate queue for each task. On the other hand, classical scheduling policies such as EDF require a single queue for all tasks and, hence, require O(1) scheduling cost. Striegel and Manimaran introduced the Enhanced Distance Based Priority (EDBP) algorithm that offers a similar performance as the DBP-M approach but maintains a fixed scheduling cost [55]. The EDBP algorithm schedules jobs by combining the DBP state of a task with the *laxity* of a single job, i.e., the remaining time until the deadline. The simulations showed that the proposed approach achieved a similar dynamic failure performance as the DBP-M approach, while keeping the scheduling cost close to that of the EDF algorithm.

Wang et al. investigated the possibility of combining DBP with scheduling policies other than EDF [56]. The motivation behind this approach is that DBP combined with EDF considers the distance to the failure state only, regardless of the distribution of missed and met deadlines. For example, consider two tasks under 25 constraint which have the following μ-patterns: 00001 and 10000. Both patterns correspond to a failure state and the DBP will assign the same priority (0) to both tasks. However, in order to exit the failure state, the task with μ-pattern 00001 needs one met deadline, while the task 10000 needs two. Wang et al. introduced integrated DBP (IDBP) as a remedy approach that overcomes the described drawback of the classical DBP. The IDBP uses a priority assignment scheme that takes into account not only the distance to the dynamic failure state but also the distance to exit out of a failure state. The evaluation of the approach showed that IDBP could effectively reduce the probability of dynamic failure under overload conditions in comparison with classical DBP.

**Figure 4:**
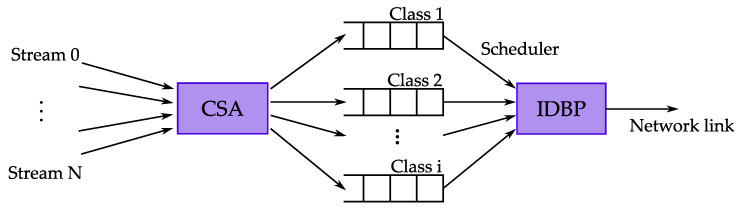
System model for the CSA. Modified from [57].

The further work of authors Chen et al. extends the EDBP algorithm by introducing the Class Selection Algorithm (CSA) [57]. The authors study the trade-off between scalability and QoS granularity. QoS granularity is a metric introduced in [58] that corresponds to the variation of the achieved and required QoS for each task. The main idea of the CSA is to provide a mechanism for classifying the tasks into a predefined number of classes, i.e., queues. Using a fixed number of classes improves the scalability at the cost of QoS granularity and, consequently, the balance between scalability and QoS granularity can be achieved. Assigning the priorities is performed on a per-class basis rather than a per-job basis and, therefore, some tasks within the class may violate their individual nm constraints. The system model for CSA is shown in Figure 4. The first step is to select a class for every task according to CSA. The entities of the classes are then scheduled according to the IDBP.

**Figure 5:**
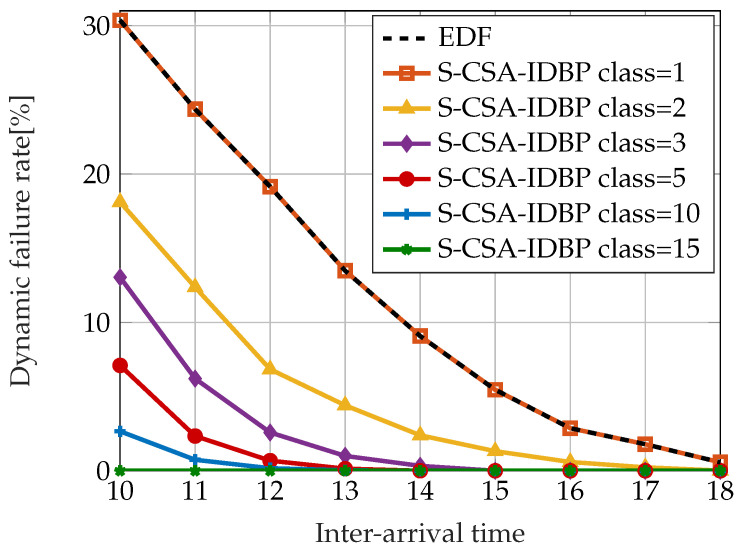
A comparison of EDF, IDBP, and S-CSA with different class numbers. Data taken from [57].

There are two different variants of the CSA with regard to the policy of assigning tasks to classes: static CSA (S-CSA) and dynamic CSA (D-CSA). In static CSA, a task is assigned to a class according to the task’s required QoS, where each class corresponds to a fixed interval of QoS value. The dynamic CSA balances the number of tasks in each queue and dynamically adjusts the bounds of QoS values of each class. Both S-CSA and D-CSA were evaluated with respect to the dynamic failure rate and compared with the IDBP and EDF approach. Figure 5 depicts the results of comparing the failure rate achieved by the S-CSA-IDBP approach with the EDF and the IDBP schedulers. The simulated system consists of 15 tasks. The *m* and *k* values are generated randomly according to uniform distribution and the tasks follow the Poisson distribution. The service time for all of the jobs equals 1. The results show that S-CSA-IDBP with one class performs the same as EDF, which is the expected behavior since it is equivalent to EDF with 15 tasks and 1 queue. On the other hand, S-CSA-IDBP with 15 classes performs the same as IDBP. The balance between the properties of the EDF and IDBP approach can be achieved by tuning the number of classes used in CSA.

Another enhancement of the classical DBP approach is the matrix-DBP, introduced in [59]. The algorithm enhances the priority assignment scheme of DBP using the minimum number of deadline misses that may occur during the service time of jobs from the other tasks. In that way, a lower bound of the actual number of deadline misses is adopted. The information about the minimum number of deadlines a task *i* can miss while task *j* is served is contained in a matrix called the mutuality matrix. The scheduler operates in the following way: each time a job needs to be scheduled, the DBP priority is updated by subtracting the corresponding matrix element. In the experiments, it is shown that matrix-DBP outperforms the classical DBP in terms of the deadline miss ratio and reduces the probability of the failure state. This approach introduces very low computational cost since it relies only on accessing the elements of a matrix.

Further research regarding the matrix-DBP approach is described in [60]. The authors introduce the equivalent matrix-DBP which extends the matrix-DBP by considering aperiodic tasks.

Another approach for scheduling real-time systems with nm constraints is presented by West and Schwan in [61]. The authors investigated the scheduling of multiple frames in multimedia streams and developed the Dynamic Window-Constrained Scheduling (DWCS) algorithm. This algorithm is designed to limit the number of late frames (jobs) in accordance with their loss and delay constraints. The priorities of frames to be transmitted are assigned according to two attributes: deadline and loss tolerance. Using the notation in accordance with nm constraints, the loss tolerance can be specified as value (m−n)/m. Note that the lower value of the loss tolerance corresponds to a state closer to dynamic failure; therefore, the scheduler gives precedence to the job of a task with the lowest loss tolerance. The jobs of the same task all have the same loss tolerance, and they are scheduled in order of arrival. When a job misses its deadline, the loss tolerance of all other jobs of the same task is reduced; thus, the priority of that task is increased. Conversely, if a job is scheduled before its deadline, the loss tolerance of the remaining jobs of the task is increased. Since the loss tolerance values of the tasks must be adjusted every time a task is serviced or not serviced within the required period, each step of the DWCS requires O(N) computation time.

In [62], West and Poellabauer describe a modified version of the DWCS algorithm that can guarantee the fulfillment of nm constraints. In this approach, the process model is restricted to implicit deadlines $D_{i}=T_{i}$ and the same value of initial loss tolerance. The authors proved that under these restrictions, the DWCS could guarantee that the weakly hard constraints will be satisfied, i.e., there will be no more than m−n missed deadlines for every *m* jobs. Moreover, the DWCS can guarantee a bounded delay of service for real-time tasks even when the scheduler is overloaded [63].

In [64], Bernat and Burns addressed the problem of deciding online which jobs should miss their deadline in order to increase the slack time available for the execution of aperiodic tasks. Their approach is based on dual priority scheduling (DPS) [65]. The main idea of DPS is to retain the predictability of hard tasks while stimulating the execution of aperiodic (soft) tasks. The DPS requires three distinct priority bands: lower, middle, and upper bands. Each hard task can execute either in the lower or upper band, while soft tasks execute in the middle band. The optimal time when a hard task must be promoted from the lower band to the upper band can be determined by offline analysis. In a system with nm constraints, the tolerance to deadline misses can be exploited to increase the responsiveness of soft tasks. This can be performed by delaying the priority promotion time by not promoting some task invocations. Several strategies for delaying the priority promotion time are presented in [64], as well as a low-cost dynamic algorithm for deciding online which tasks to promote.

Overload management techniques in real-time systems with nm deadlines were discussed in [11] in the context of recovering from failure state in control loops. The proposed method relies on an offline scheduling algorithm that reduces the equivalent utilization in overload conditions by partitioning the jobs into mandatory and optional. The partitioning is performed in such a way that nm constraints are satisfied. The mandatory jobs are scheduled with fixed priorities using the Rate Monotonic policy in a way that the deadlines of the mandatory jobs are guaranteed. Optional jobs are assigned the lowest priority and are not guaranteed to meet their deadlines. A job activated at time $aT_{i}$ is classified as mandatory if:

(7) $$a=\left\lfloor \left\lceil \frac{an_{i}}{m_{i}}\right\rceil \cdot \frac{m_{i}}{n_{i}}\right\rfloor$$

It is shown in [11] that this condition guarantees that at least $n_{i}$ out of $m_{i}$ consecutive instances are classified as mandatory; therefore, the nm constraint is guaranteed. It is important to emphasize that this classification of instances is not necessarily optimal.

Further research related to comprising a policy for determining mandatory jobs is described in the work of Quan and Hu. In [66], it is shown that the problem of finding the optimal policy for partitioning the jobs into mandatory and optional is NP-hard, as well as the problem of determining the schedulability of the resultant task set. The authors presented an optimization technique for improving the job partitioning policy presented in [11]. The main idea of this technique is to improve the schedulability of the task set by modifying the pattern of mandatory and optional jobs in order to reduce the execution interference between tasks, i.e., the amount of time in which preemption between mandatory jobs can occur. The authors proposed a novel technique for determining the interference among tasks, based on the General Chinese Remainder Theorem (introduced in [67]). The execution interference is used for computing the objective function for the optimization process. The authors suggested two optimization approaches for obtaining the job partitioning pattern: simulated annealing and genetic algorithm. The experiments showed that the suggested heuristic approach brings significant improvements to the schedulability, especially in overload conditions.

**Figure 6:**
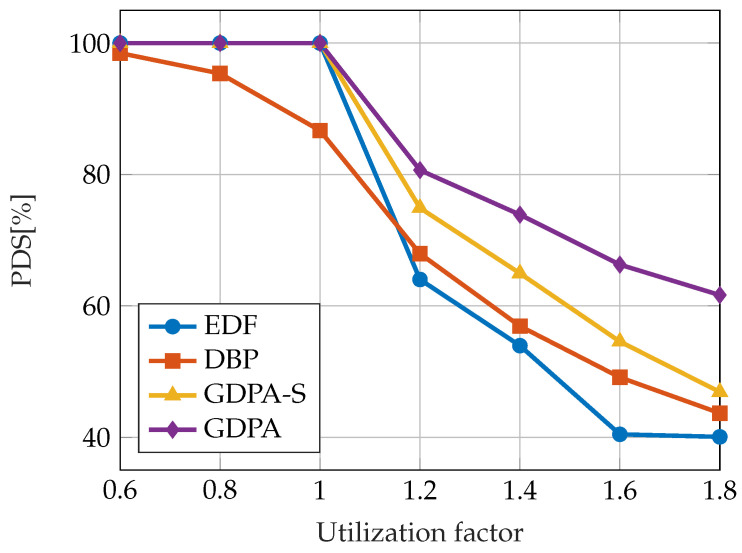
A comparison of PDS obtained by the GDPA, GDPA-S, DBP, and EDF approaches. Data taken from [68].

In one particular paper [68], the guaranteed dynamic priority assignment (GDPA) scheme is presented, which is designed for maximizing the QoS of tasks with nm constraints while providing a bounded probability of violating the constraints when the system is underloaded. The order of active jobs is determined with respect to both the distance to the failure state and the earliest deadline. In every step of the algorithm, the feasibility of the constructed job sequence is tested. It is proven that GDPA provides scheduling optimality in the sense that GDPA satisfies all task deadlines when the system is under-loaded, as does the EDF algorithm, and it is guaranteed that no dynamic failure will occur. Due to the required online feasibility tests, the computational cost of the GDPA algorithm is $O(N^{2})$. For applications that do not tolerate high computational costs, a low-cost version of GDPA is presented - simplified GDPA (GDPA-S). This approach introduces O(N) computational cost. The results of evaluating the proposed approaches are shown in Figure 6. The metric used for comparison is the probability of deadline satisfaction (PDS), which is defined as the ratio of deadline satisfaction to job releases and it corresponds directly to the QoS. The tasks were randomly assigned different nm constraints. The EDF, GDPA, and GDPA-S support 100% PDS when the total utilization demand is less than or equal to 1. In overload conditions, GDPA and GDPA-S achieved the highest PDS.

In the context of cyber-physical applications, the researchers have applied the weakly hard real-time constraints to sporadic tasks in order to reduce the interference of sporadic tasks to other tasks [16]. A new scheduling algorithm is introduced, namely the job-class-level scheduler (JCLS). The main idea is to assign a class to the sporadic job, depending on the number of previously met deadlines. The performance of the proposed method is compared to other methods from the literature, RTO with the rate-monotonic period assignment and weakly hard schedulability analysis ([25]) with respect to the task set schedulability. JCLS showed significantly higher schedulability ratios than other considered methods.

In [69], a scheduling algorithm, namely Global Emergency-Based Scheduling (GEBS), that aims to increase the schedulability ratio of weakly hard real-time tasks is presented. Priority assignment is based not only on the run-time properties of jobs, but also on emergency classes of all tasks, making this approach a global priority allocation scheme. The algorithm for determining an emergency class of a task relies on feasibility tests in order to increase the schedulability of the given task set. The GEBS approach is compared to JCLS [16] and the scheduling approach presented in [66], and it is shown that GEBS yields higher schedulability ratios and a better performance in the sense of run-time overhead.

### 4.2. Scheduling Approaches for the Skip-Over System Model

The authors of the skip-over model have presented several online scheduling algorithms which implement the skippable method for reducing permanent overload. The technique of reducing the permanent overload is known in the literature as the job-skipping method [70].

The results of evaluating the job-skipping algorithms are derived under the assumption of a deeply red condition, defined in Section 5.2.

The authors of the skip-over model have proposed and analyzed two online scheduling algorithms that exploit skips: the Red Tasks Only (RTO) algorithm and the Blue When Possible (BWP) algorithm. In the following text, the job-skipping algorithms are described. The Red Tasks Only (RTO) algorithm decreases the system load by always skipping the blue jobs, regardless of their impact on the system load. Red jobs are scheduled according to the EDF algorithm. It was proven in [47] that the RTO algorithm is optimal under the deeply red condition in the sense that all feasible sets are schedulable using RTO. It is worth noting that under RTO, the distance between two consecutive skips is exactly $m_{i}$ ($s_{i}$) periods. The Blue When Possible (BWP) algorithm allows the blue jobs to execute if there are no active red jobs in the system. The red jobs are scheduled according to the EDF algorithm, whereas blue jobs can be scheduled according to arbitrary scheduling policies, including the EDF, the latest deadline first, etc.

Scheduling task sets with skippable jobs was further researched by authors Marchand and Silly-Chetto [71,72,73]. The authors have introduced the Red Tasks as Late as Possible (RLP) algorithm, which stimulates the execution of blue jobs by executing them at idle times of a preliminary schedule considering only the red jobs. Idle time refers to the time span in the schedule where there are no red jobs in the ready state. The RLP algorithm is specified by the following characteristics:

- If there are no blue jobs in the system, red jobs are scheduled as soon as possible according to the EDF;
- If blue jobs are present in the system, red jobs are processed as late as possible and blue jobs are processed in the idle time of red jobs.

Blue jobs can be scheduled in idle time by any arbitrary scheduling scheme. The mechanism for determining the idle times of the red jobs relies on the Earliest Deadline as Late as Possible (EDL) algorithm. The EDL algorithm was introduced in [74], and it was initially designed to minimize the response time of soft aperiodic tasks by dispatching them in the idle times of periodic tasks. In the context of the RLP algorithm, the EDL algorithm is used for determining the latest start time for red jobs in order to maximize the slack time for dispatching blue jobs. Moreover, once the idle intervals are known, it can be decided online whether it is feasible to accept blue jobs. The online computation of available slack time is required when a blue job is released while there are no active blue jobs in the system and after the completion of a blue job, if blue jobs remain in the system [73].

In [75], Marchand and Silly-Chetto introduced the RLP with blue acceptance test (RLP/T) algorithm which enhances the QoS achieved by the RLP algorithm. The authors pointed out the risk of aborting the blue jobs before the completion as a main drawback of the RLP algorithm. Therefore, an online test should be performed in order to decide whether a blue job can be accepted for execution. The general idea of the algorithm is as follows: red jobs enter the system upon activation time, while blue jobs enter the system upon acceptance. Once a blue job is accepted, it is scheduled as soon as possible together with the red jobs. The acceptance test is based on the method presented in [76], which was initially used for performing acceptance tests of sporadic tasks occurring in an environment consisting of periodic tasks. In this context, the method is used for computing the slack time available for the execution of a blue job, i.e., the maximum amount of time during which the job could not be scheduled without missing its deadline. The complexity of performing the acceptance test is $O(\left\lfloor \frac{D_{\max}}{T_{\min}}\right\rfloor N+B\left(t\right))$, where *N* is the number of periodic tasks, $D_{\max}$ is the longest deadline, $T_{\min}$ is the shortest period, and B(t) denotes the number of blue jobs active at time *t* [72].

**Figure 7:**
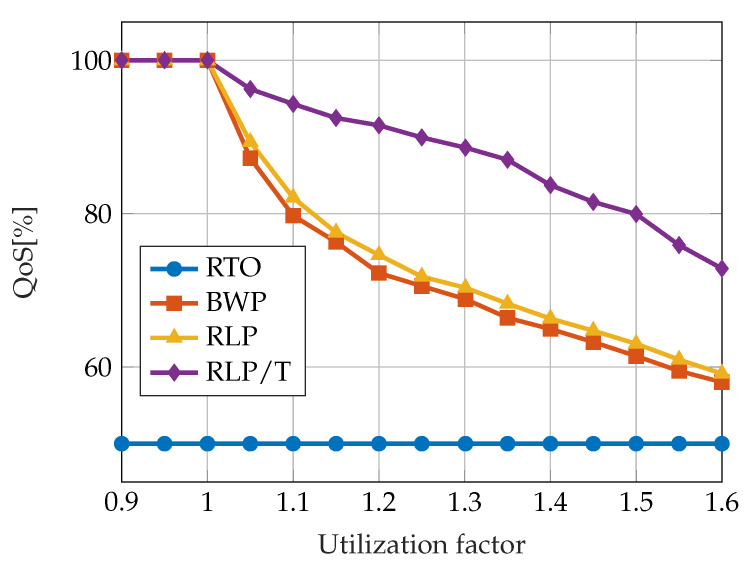
A comparison of job-skipping algorithms according to QoS metric. Data taken from [73].

A comparison of the QoS achieved by the described algorithms is given in Figure 7. The results were obtained by simulating the execution of 50 periodic task sets, each consisting of 10 tasks with equal skip factors, $m_{i}=2$. The results are compared with respect to the total system load. The system load is varied in the interval [0.9,1.6] by tuning the worst-case execution time of the tasks. The QoS of the RTO algorithm equals 50% regardless of the system load. The performance of the RLP and BWP algorithms is similar but significantly worse than RLP/T.

**Figure 8:**
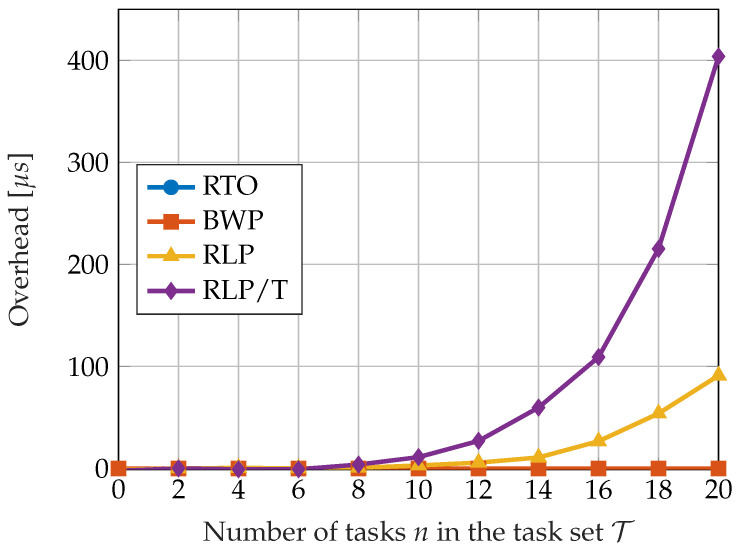
A comparison of job-skipping algorithms with respect to the dynamic overhead. Data taken from [73].

Figure 8 shows the comparison of the overhead introduced by the described algorithms with respect to the total number of tasks in the set. The overhead corresponds to the amount of CPU time spent on performing scheduling operations. The measurements were performed over a simulation time of 1000 s. The algorithms BWP and RTO do not introduce any overhead, as they do not require the online computation of slack time or performing schedulability tests. The results obtained by the RLP algorithm are a consequence of computing the idle times according to the EDL. The overhead introduced by the RLP/T scheduling algorithm significantly increases with the number of tasks. This is due to the fact that the EDL idle times must be computed every time a blue job is released or completed.

The described algorithms consider a system that consists solely of skippable periodic tasks. The problem of combining skippable periodic tasks with soft aperiodic requests was addressed in the work of Caccamo and Buttazzo [77]. Since aperiodic tasks must be served as soon as possible in order to minimize their response time, the authors investigated the possibility of exploiting skips in order to enhance the responsiveness of aperiodic tasks. In the proposed technique, the RTO and BWP algorithms were integrated with the Total Bandwidth Server (TBS) algorithm [78]. The TBS algorithm provides an online mechanism for scheduling aperiodic requests while guaranteeing the execution of periodic tasks.

In their further work [79], Buttazzo and Caccamo introduced a mechanism for tuning the deadline assignment algorithm presented in [77] to balance performance and complexity. Moreover, they described how the TBS can be improved up to optimality in order to achieve the minimum response time.

In [80], the performance of the BWP and RLP algorithms was improved by introducing a hyper-heuristic approach for optimizing the QoS. More precisely, heuristics evolved by genetic programming were used for assigning priorities of blue jobs. The presented approach can improve the QoS by up to 15% in overloaded conditions in comparison with RLP and BWP. Moreover, the evaluation of the priority assignment heuristics is computationally efficient, and it does not introduce overhead.

### 4.3. Scheduling Approaches for Generalized Weakly Hard Real-Time System Model

There are very few scheduling approaches that support weakly hard constraints other than the nm constraint. In [4], the authors of the weakly hard real-time system model discuss a method for optimal priority assignment for task sets under weakly hard constraints. The optimal priority assignment can be found by applying the partition method and it is based on the property that if a task is schedulable under weakly hard constraints at a priority *p*, then it is also schedulable under any priority higher than *p*. The algorithms assign an initial priority to each task, e.g., Deadline Monotonic, and check whether the lowest priority task meets its constraints. If this is not the case, then another task is assigned the lowest priority and the process is repeated. The procedure of finding the optimal priority assignment is finished when a priority assignment scheme is found such that all tasks are schedulable or when no task is found to be schedulable at a given priority level. In the latter case, the task set is not feasible.

A scheduling algorithm that supports both the nm and nm constraints is the Bi-Modal scheduler [81]. The Bi-Modal (BM) scheduler is an online scheduling framework that considers both periodic and non-periodic tasks and is constrained to implicit deadlines. The scheduler is based on two different scheduling modes. In normal mode, the jobs are scheduled according to an arbitrary scheduling policy that may take into account the history of missed and met deadlines. If a dynamic failure is likely to occur, the scheduler switches into panic mode, for which it is guaranteed that a job will meet its deadline. When a job is released, it is assigned one of two states according to the criticality of meeting its deadline. A non-critical state is assigned if the job can miss its deadline without jeopardizing the satisfaction of the weakly hard constraint of the corresponding task. Non-critical jobs are scheduled by a scheduler in normal mode. If the job must meet its deadline to satisfy the weakly hard constraint, it is assigned a critical state and it is scheduled in panic mode.

The schedulability tests that guarantee that all critical jobs can be scheduled in panic mode are based on a worst-case response time analysis. The authors proved that a system would never produce a dynamic failure if critical jobs are promoted to panic mode at least $R_{i}$ time instances before their deadline [81].

The state of a released job is determined through a criticality function. The criticality function $\delta^{\lambda }\left(\alpha \right)$ determines the number of consecutive deadlines that can be missed without violating the weakly hard constraint λ, based on the μ-pattern α of the task. In [81], several criticality functions are defined for the four types of weakly hard constraints. If $\delta^{\lambda }\left(\alpha \right)=0$, the released job is in the critical state and it must be scheduled in panic mode. This can be conducted as soon as the critical job is released (immediate panic mode) or with some delay if there is slack time available (delayed panic mode). The panic mode is implemented as a fixed priority mechanism, where the priority of critical jobs is strictly higher than non-critical jobs. The authors suggest assigning priorities according to the optimal priority assignment described in the previous text. The non-critical jobs can be scheduled according to any scheduling policy. It can be useful to apply scheduling policies that keep track of the tasks’ μ-patterns and thus avoid entering panic mode.

**Figure 9:**
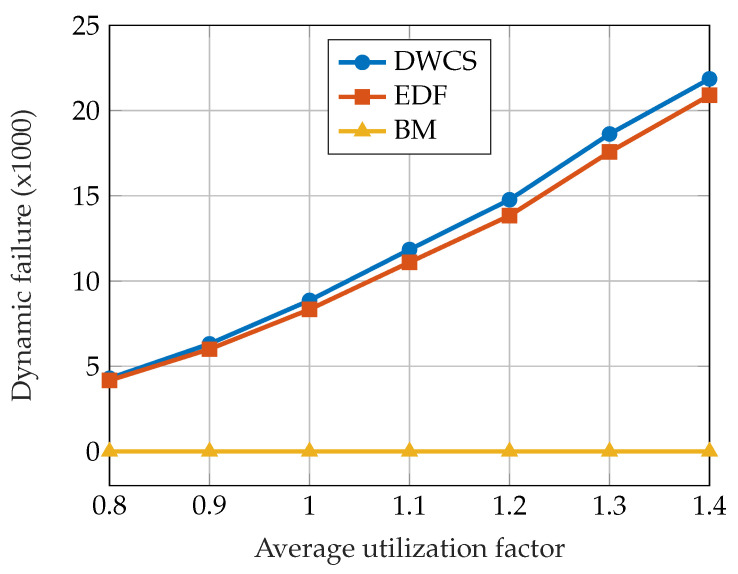
A comparison of the number of dynamic failures for DWCS, EDF, and BM scheduling algorithms. Data taken from [81].

The authors evaluated the suggested approach by analyzing the number of dynamic failures and comparing it with other approaches: the EDF and the DWCS approach [62]. The performance of the scheduling algorithms was tested on a large number of synthetically generated task sets, with the average utilization factor in the interval [0.8,1.4]. The EDF scheduler was used for scheduling jobs in the normal mode under the BM scheduler. The results of the evaluation are shown in Figure 9. No dynamic failures occurred under the BM scheduling algorithm, which is expected behavior.

Another approach for the guaranteed scheduling of task sets under weakly hard constraints is described in [82]. Two algorithms, the Meet any algorithm (MAA) and the Meet row algorithm (MRA) are introduced for scheduling tasks under the nm and nm constraints, respectively. Both algorithms aim to avoid dynamic failure by increasing the priority of a task at risk of entering a failure state in advance. The computation of priority $\pi_{ij}$ for each job is based on the combination of two expressions:

(8) $$\pi_{ij}=\alpha T\left(\tau_{i}\right)+\beta C\left(\tau_{i}\right)$$

where $T(\tau_{i})$ is a priority assignment expression that corresponds to any arbitrary algorithm (e.g., EDF) and $C(\tau_{i})$ is an expression that guarantees the weakly hard deadlines. The expression $C(\tau_{i})$ is different for the nm and nm constraints. α and β are positive real numbers that determine the weight of the constraints $T(\tau_{i})$ and $C(\tau_{i})$. In the context of this approach, a larger value of $\pi_{ij}$ corresponds to a higher priority value. The authors propose a technique for calculating the value of $C(\tau_{i})$ based on the number of met deadlines in the μ-pattern such that the nm constraint is satisfied. The computation cost of the proposed technique for determining $C(\tau_{i})$ is O(m). In [83], the authors propose a technique for determining $C(\tau_{i})$ for the MRA algorithm in O(1) complexity.

**Figure 10:**
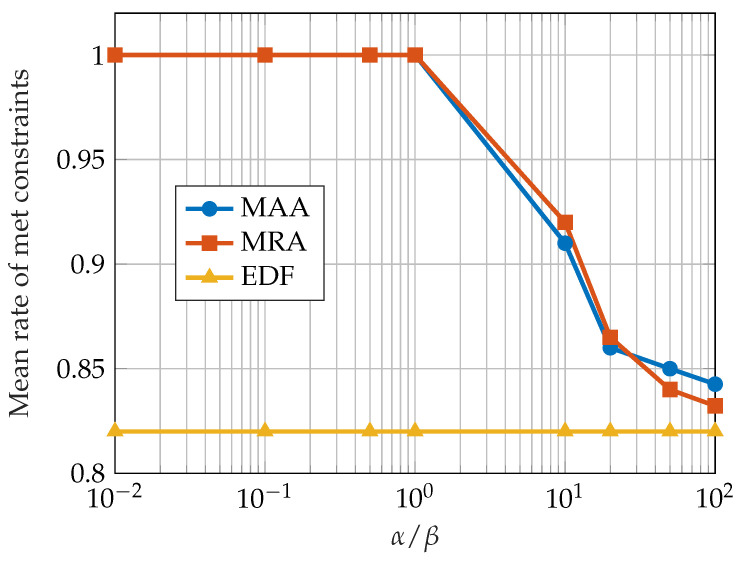
Mean rate of met deadlines obtained by the MRA, MAA, and EDF algorithms. Data taken from [82].

Figure 10 shows the mean rate of met constraints achieved by the MRA and MAA approach with different values of α/β for a CPU utilization of 1.3. The results are compared with the EDF approach, which obtained a constant rate of met constraints since the CPU utilization is also constant. When the α/β ratio is less than 1, the guaranteed behavior is emphasized; the mean rate of met constraints approaches 1.

Table 3 provides a summary of the scheduling algorithms reviewed in this section, including algorithms for the (m,k)-firm, skip-over, and generalized weakly hard real-time models. The algorithms are classified concerning whether they guarantee that no dynamic failures will occur, or they aim to minimize the probability of dynamic failure (guaranteed or best-effort). The categorization of the corresponding priority assignment policies into fixed priority and dynamic priority assignments is denoted by *FP* and *DP*, respectively. The complexity of the priority assignment for each algorithm is discussed, taking into account the number of tasks *N*, the maximum length of the μ-pattern $M_{\max}$, and other algorithm-specific parameters. Additionally, the main advantages of the described algorithms are listed.

## 5. Schedulability Analysis for Weakly Hard Real-Time Systems

This section describes the approaches for determining the schedulability for the task models described in Section 3. Additionally, the schedulability conditions derived for specific scheduling algorithms are presented.

### 5.1. Schedulability Analysis for (m,k)-Firm System Model

Given a set of *N* periodic tasks with nm constraints, the task set is said to be schedulable if it is possible to find a scheduling algorithm that allows for meeting the nm constraints of all tasks. The scheduling algorithm typically yields a pattern that defines which jobs are mandatory, i.e., the deadline must be met, and which jobs are optional, i.e., the deadline can be missed. It is proven that the problem of deciding whether there exists a pattern of mandatory and optional deadlines for each task in a set T such that T is schedulable is NP-hard in the strong sense [66]. Moreover, given a pattern of mandatory and optional deadlines for each task in T, the problem of determining whether T is schedulable is also NP-hard [66]. The schedulability analysis of weakly hard real-time systems typically relies on sufficient conditions derived for specific scheduling algorithms.

A sufficient condition for the schedulability of task sets under nm constraints for a specific case, where task periods are harmonic, is derived in [66]. For tasks with harmonic periods, each period is an integer multiple of shorter periods. The tasks are assumed to have implicit deadlines, i.e., $D_{i}=T_{i}$.

**Theorem** **1.** *Given two task sets T and $T^{\prime }$ with $T_{i}^{\prime }\leq T_{i}$, $C_{i}^{\prime }=C_{i}$, $n_{i}^{\prime }=n_{i}$, $m_{i}^{\prime }=m_{i}$, and $T_{j}^{\prime }$ divides $T_{i}^{\prime }$ if $T_{j}^{\prime }\leq T_{i}^{\prime }$. With the given nm constraints, T is schedulable if:*

(9) $$\sum_{j\leq i}\frac{l_{ij}C_{j}}{T_{i}^{\prime }}\leq 1$$

*where $l_{ij}$ is the maximum number of mandatory jobs during any time interval of length $T_{i}^{\prime }$.*

This condition is an extension of the research of Han and Tyan [84], where the authors introduced a polynomial-time algorithm for testing the schedulability of a hard real-time system under fixed priorities. This approach is based on mapping each task in the set to a new task such that the new period is less than or equal to the original period, while the computation time remains the same. The mapping of the task periods is conducted in such a way that the resulting task set must be harmonic. It is proven in [84] that if the new harmonic task set is schedulable, so is the original task set. Given the technique described in [84], a corresponding harmonic task set can be found and the schedulability of the task set can be tested according to Theorem 1. An implementation of this approach takes $O(N^{3}m\log N)$ computation time, where $m=\max m_{i}$ and *N* is the number of tasks.

### 5.2. Schedulability Analysis for the Skip-Over System Model

The feasibility problem in the context of the skip-over task model amounts to the problem of finding deadlines to be skipped such that no weakly hard constraint m−1m is violated and the remaining jobs can be scheduled to meet their deadlines. Koren and Shasha proved in [47] that determining whether a set of periodic occasionally skippable tasks is schedulable is NP-hard in the weak sense. This result corresponds to a general case where the jobs can be either red or blue when they are released. However, schedulability conditions have been derived by assuming a specific case, i.e., the deeply red condition, where all jobs are released simultaneously and the initial state of each job is red. The deeply red condition is the worst-case assumption for the skip-over model; if a task set is schedulable assuming the deeply red condition, it is also schedulable without this assumption. Koren and Shasha have introduced a necessary condition for the schedulability of a set of skippable tasks [47], which is given in Lemma 1.

**Lemma** **1.** *Given a set $T=\{\tau_{i}{\left(T_{i},C_{i},m_{i}\right)}_{i=1}^{N}\}$ of periodic tasks that allow skips, then*

(10) $$\sum_{i=1}^{N}\frac{C_{i}\left(m_{i}-1\right)}{T_{i}m_{i}}\leq 1$$

*is a necessary condition for the feasibility of T, because that sum expresses the utilization based on the computation that must take place.*

The sufficient condition was constructed through the processor demand criteria that a set of periodic tasks is schedulable if and only if for all intervals L≥0 [85]:

(11) $$L\geq \sum_{i=1}^{N}\left\lfloor \frac{L}{T_{i}}\right\rfloor C_{i}$$

The right-hand side of the inequality is the processor demand in the interval [0,L], where each task $\tau_{i}$ releases exactly $\left\lfloor L/T_{i}\right\rfloor$ jobs that require a processor time of $C_{i}$ units. If the cumulative processor demand in the interval [0,L] is less than *L*, all of the jobs will complete within their deadlines. Koren and Shasha extended this condition to the skip-over system model and they introduced the sufficient schedulability condition:

**Lemma** **2.** *Given a set $T=\{\tau_{i}{\left(T_{i},C_{i},D_{i},m_{i}\right)}_{i=1}^{N}\}$ of periodic tasks that allow skips, then*

(12) $$L\geq \sum_{i=1}^{N}D\left(i,\left[0,L\right]\right)\;\forall L\geq 0$$

*is a sufficient condition for the feasibility of T, where D(i,[0,L]) is the CPU time demanded by the task $\tau_{i}$ in the time interval [0,L].*

(13) $$D\left(i,\left[0,L\right]\right)=\left(\frac{L}{T_{i}}-\frac{L}{T_{i}m_{i}}\right)C_{i}$$

It is worth noting that the maximum value for *L* to be checked is the hyperperiod $H=\mathrm{lcm}(T_{1},T_{2},\ldots ,T_{N})$ instead of $\mathrm{lcm}(m_{1}T_{1},m_{2}T_{2},\ldots ,m_{N}T_{N})$ because the algorithm skips at the end of $T_{i}m_{i}$ intervals. Therefore, if the schedule is feasible in the interval [0,H], it is feasible in any interval.

### 5.3. Schedulability Analysis for the Generalized Weakly Hard Real-Time System Model

The schedulability tests for generalized weakly hard constraints are based on verifying whether the μ-pattern obtained by executing the jobs with their worst-case execution times satisfies the given weakly hard constraint. In the general case, the μ-pattern is an infinite binary sequence, which is not suitable for performing schedulability tests. In [4], the authors proved that the μ-pattern is *closed*, i.e., made up of cyclic repetitions of a word, if the total utilization of the task set is no greater than one. Therefore, the offline schedulability analysis presented in [81] is only possible for task sets where U≤1. The schedulability test is extended to the systems where blocking factors between tasks must be taken into account, e.g., the priority ceiling protocol [86]. In order to construct the μ-pattern, the worst-case finalization time $F_{ij}$ for each job must be computed. $F_{ij}$ is the minimum t>0 that makes the following equation hold:

(14) $$t=kC_{i}+{\mathrm{Intf}}_{i}\left(t\right)+{\mathrm{Idle}}_{i}\left(S_{i}\left(j\right)\right)$$

where ${\mathrm{Intf}}_{i}\left(t\right)$ is the interference from higher priority jobs up to time *t* and ${\mathrm{Idle}}_{i}\left(S_{i}\left(j\right)\right)$ is the amount of time the processor can be used by tasks of lower priority than $\tau_{i}$ within a period of time [0,t). $F_{ij}$ can be calculated by a recurrence formula given in the following equation:

(15) $$\left\{\begin{array}{c} t_{0}=0\\ t_{N+1}=kC_{i}+{\mathrm{Intf}}_{i}\left(t\right)+{\mathrm{Idle}}_{i}\left(S_{i}\left(j\right)\right) \end{array}\right.$$

The recurrence finishes when $t_{N+1}=t_{N}$. The μ-pattern is constructed according to the expression in (5) and it is evaluated whether the μ-pattern satisfies the given constraint. The drawback of this approach is that it relies on the assumption that the initial offsets of all tasks are known, which may be too strict.

The results that allow relaxing the requirement of knowing the initial system state are achieved in the research of overload conditions in weakly hard real-time systems. The proposed approach relies on the method introduced in [87], where the worst-case behavior of a system is analyzed as a combination of typical behavior, i.e., execution of periodic tasks, and sporadic overload. This approach is applied to weakly hard real-time system analysis in [22,88]. The main idea of the proposed approaches consists of a two-step method:

1. Check whether the system is schedulable under the typical behavior using classical analysis;
2. Check if the given nm constraint is satisfied in overload conditions.

The limitation of knowing the explicit initial system state is overcome in [25]. The authors provide a generalized framework for scheduling periodic tasks under weakly hard constraints on a uniprocessor system. The proposed approach is based on mixed-integer linear programming (MILP) problem formulation. The framework has been extended to real-time systems with shared resources and task sets with release jitter, i.e., the maximum possible delay of actual activation time of a job with respect to ideal periodic activation. In [48], this work is extended by providing an analysis for job-kill strategy, i.e., terminating a job when it misses its deadline instead of continuing the execution of the job (as in the job-continue strategy).

## 6. Applications of the Weakly Hard Real-Time Model in Control Systems’ Design

This section brings an overview of the existing approaches for control and scheduling co-design. Research in this area is focused on two main problems:

- Stability analysis: determining timing constraints for control tasks that ensure control loop stability;
- Optimal control system design: leveraging the weakly hard real-time constraints to reduce system utilization in overload conditions while aiming to maximize control performance.

Before presenting the most important research results from this area, basic concepts from control theory are outlined.

### 6.1. Overview of Basic Concepts from Control Theory

**Figure 11:**
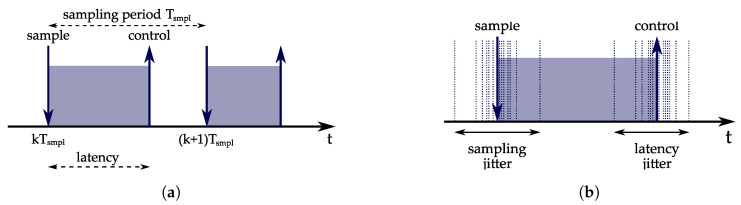
An illustration of control loop timing characteristics. (**a**) Input-output latency and sampling period. (**b**) Sampling and latency jitter.

A fundamental component of a control system is a control loop. A control loop performs three functionalities: sensor sampling, control algorithm computation, and sending output commands to actuators. The functionalities of the control loop are usually executed periodically with a constant sampling period determined by the process dynamics. Figure 11 illustrates the timing constraints of a control loop. Ideally, sampling and command output occur at exact time instants. The sampling time $t_{\mathrm{smpl}}$ is determined by the *sampling period*, $T_{\mathrm{smpl}}$: $t_{\mathrm{smpl}}=kT_{\mathrm{smpl}}$. In real-world applications, there are deviations from the sampling period, i.e., sampling jitter. The command output time depends on the input-output latency, also referred to as the control delay, which should be as small as possible. Typically, there are deviations from the estimated input-output latency, i.e., latency jitter. An increase in the input-output latency or jitter decreases the stability margin of the control system. In [2], it is described how the controller can be designed to actively compensate for the sampling jitter and reduce the effects of timing nondeterminism by recomputing the controller parameters. However, in control systems that are more complex, it is not suitable to apply such techniques and it is more appropriate to rely on the mechanisms from real-time scheduling theory. In computer-driven control systems, the control loops are implemented as real-time tasks. From the real-time scheduling perspective, the objective is to configure the tasks and design a scheduling algorithm such that the input-output latency and jitter are minimized. On the other hand, from the control theory perspective, the goal is to optimize control performance. A commonly used metric for measuring control performance is the *performance index*, which is defined as follows [89]:

**Definition** **8**. *A performance index is a quantitative measure of the performance of a system and is chosen so that emphasis is given to the important system specifications.*

The performance index is computed through one of three measures:

- Integral squared error, ISE: $\int_{0}^{t}e^{2}\left(t\right)dt$;
- Integral absolute error, IAE: $\int_{0}^{t}\left|e\left(t\right)\right|dt$;
- Integral time-weighted absolute error, ITAE: $\int_{0}^{t}t\left|e\left(t\right)\right|dt$.

where e(t) is the error signal that corresponds to the deviation between the output of the control system and the desired output. The methods for control systems’ design typically aim to minimize performance index.

In many practical applications, less precise metrics are used for measuring control performance due to their simplicity and applicability to real-world scenarios. A metric that takes the error signal e(t) into account is the steady-state error:

(16) $$E_{ss}=\lim_{t\to -\infty }e\left(t\right)$$

**Figure 12:**
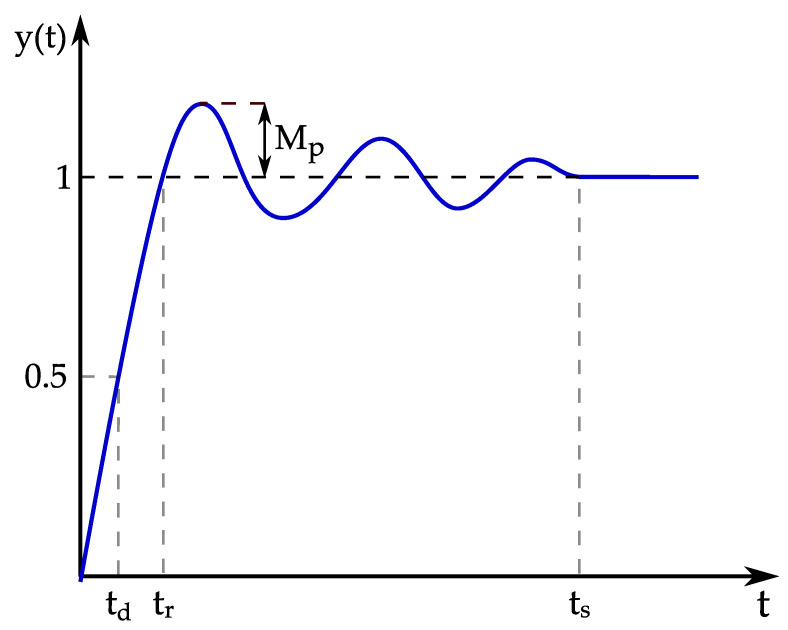
An illustration of performance metrics analyzed through the step response: delay time $t_{d}$, rise time $t_{r}$, overshoot $M_{p}$, and settling time $t_{s}$.

There are several metrics that are analyzed through the step response of the system: delay time, rise time, overshoot, settling time, etc. The delay time is the time elapsed from t=0 to the time instant at which the step response reaches half of its final value. The rise time is the time required for the step response to rise from 0% to 100% of its final value (or 10% to 90%). Overshoot is defined as the deviation of the maximum value from the step value. The time required for a step response to reach the steady state is defined as the settling time. The described metrics are illustrated in Figure 12.

### 6.2. Review of the Applications of the Weakly Hard Real-Time Model in Control Systems

**Figure 13:**
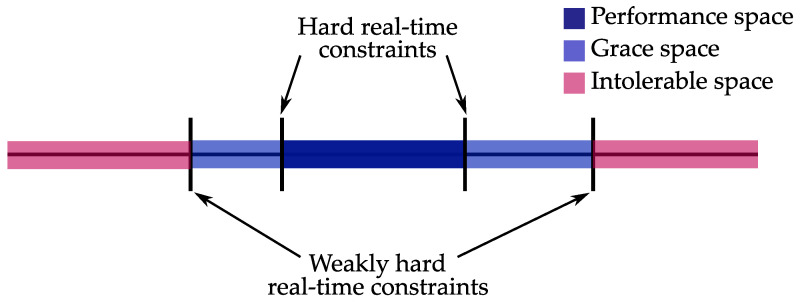
An illustration of the allowed state space.

Some of the first approaches that bind the classical control theory and real-time constraints are presented in [90,91]. These methods introduce the concept of *hard deadlines* and *allowed state space* for control systems in order to define the temporal constraints of the control system. The hard deadline for a control task is derived from the conditions of asymptotic stability of the controlled plant. In this case, the hard deadline is not associated with the period of the control task–it is the critical value of the latency jitter beyond which the system leaves its allowed state space and becomes unstable. By weakening the hard real-time constraints, the allowed state space can be split into performance space and grace space, as shown in Figure 13. The limits of performance space are determined by conventional hard real-time constraints, while the limits of the grace space are determined by weakly hard real-time constraints.

When all of the jobs of the control tasks are completed in a timely manner, the system is in the performance space. If not all of the jobs of the control tasks are completed, but the weakly hard constraint derived from stability conditions is satisfied, the system finds itself in the grace space. The performance space and the grace space comprise a tolerable space, which is bounded by the weakly hard constraint.

Initial research on resolving overload conditions in real-time control systems by relaxing hard real-time constraints was based on adapting the sampling period of a controller (task periods) such that all of the tasks are schedulable and the system performance index is optimized [92]. The authors present an algorithm that determines the task periods such that the tasks are schedulable under EDF and Rate Monotonic Scheduling (RMS). The novelty of this approach is that it does not consider task schedulability only, but also optimizes the performance of the control system. Shin and Meissner demonstrated a method for the online application of this approach in multiprocessor systems [93].

Research presented in [94] is focused on deriving timing constraints of real-time control tasks to guarantee schedulability and control performance specifications. The control performance is measured through the steady-state error, maximum overshoot, settling time, and rise time. A heuristic approach is applied to optimize the performance and to achieve a trade-off between loop processing periods and input-output latencies.

Buttazzo et al. [95] introduced a new task model, namely the elastic task model, in order to increase the flexibility of hard real-time constraints. Under this model, task periods are given elastic coefficients, which specify the ability of the task to adjust its period within certain bounds. In overload conditions, a task can decrease its period and consequently decrease its utilization. In this way, the system can stay feasible even in overload conditions.

Since the main research problem in the area of scheduling and control co-design is to design a scheduler that optimizes the control performance while keeping the CPU utilization at a desired value, the researchers have introduced approaches that interpret the scheduler itself as a controller [96]. The controlled variable is the deadline miss ratio for the tasks and the control signal is the requested CPU utilization. A similar approach is presented for multimedia applications for controlling the QoS [97,98]. In [99], the problem of scheduling control tasks on a single CPU is formalized as a linear-quadratic (LQ) control problem, with the objective of minimizing the global control cost (sum of respective control costs of all tasks) subject to constraints determined by the schedulability conditions. This method is a nonlinear programming problem; therefore, it is too computationally demanding for implementation in embedded real-time systems since they usually have limited computation resources.

The effect of latency jitter on the stability of a control system is studied in [100]. The authors propose the notion of *jitter margin* for control tasks, which defines the amount of latency jitter that can be tolerable before the control loop becomes unstable. Moreover, the authors introduce a control and scheduling co-design method that guarantees the same performance degradation for each task under overload.

The weakly hard real-time constraints are included in the real-time scheduling and control co-design in [101]. The method presented in this work is focused on providing a mechanism that guarantees the worst-case performance requirements. It relies on the concept of an accelerable control task. Accelerable control tasks decrease the value of the performance index with every execution that is completed in a timely manner. In other words, the more executions of the control task, the better the control performance. In the proposed method, the Bellman optimality principle is applied for designing accelerable control tasks– the goal is to find a control sequence that minimizes the performance index, assuming the worst-case distribution of timely executed and skipped jobs, which is referred to as the worst-case execution sequence. The worst-case execution sequence corresponds to a μ-pattern that is constructed assuming the worst case from the schedulability condition (7), i.e., only mandatory jobs at invocation count $a=\lfloor \left\lceil \frac{an_{i}}{m_{i}}\right\rceil \cdot \frac{m_{i}}{n_{i}}\rfloor$ will meet their deadlines. Therefore, mandatory jobs guarantee stability, while optional jobs improve performance if they are executed on time. In [31], this approach is applied to networked control systems that share a single Control Area Network (CAN). Another approach is to find a maximum number of consecutive deadline misses that can be tolerated, which was studied in [102]. The authors applied the presented methodology to a case study of the pitch control of an aircraft.

Hertneck et al. [103,104] analyzed the stability of a nonlinear control system. In this work, weakly hard real-time constraints were used for modeling control systems that occasionally run in open-loop due to failures in the feedback channel a failure in the control loop execution corresponds to a deadline miss. Control loop execution failures are constrained through nm constraints. The presented approach is based on a technique presented in [105], which constrains the maximum number of consecutive control loop execution failures (maximum number of successive failures). The key difference is that the technique presented in [103] uses weakly hard real-time constraints to derive the stability conditions instead of the maximum number of control loop execution failures, which results in less conservative stability conditions.

Pazzaglia et al. [106] proposed a strategy for optimal controller design that explicitly takes into account the possibility of missing deadlines– the Deadline-Miss-Aware Controller (DMAC). The proposed method considers task periods that are shorter than the worst-case response time of the tasks and estimates deadline miss occurrences according to probabilistic analyses of the task execution times. Three different strategies for handling deadline misses are considered in the analysis: aborting the job that missed its deadline (job-kill), allowing the job to continue but skipping the next job, and letting all jobs execute until completion but limiting the capacity of the queue that contains pending jobs. In [107], a stability analysis under the $\bar{\langle n\rangle }$ weakly hard real-time constraint is performed. The authors analyzed several strategies for handling a deadline miss. From the control perspective, upon a deadline miss the control signal can either be set to zero or set to a value from the previous period. On the other hand, from the task management perspective, a job that missed its deadline can either be aborted (job-kill strategy) or allowed to continue (job-continue strategy). The stability criteria are derived for each of these strategies.

Recent work by Linsenmayer et al. [13] is the first study of linear control systems stability subject to weakly hard real-time constraints that considers not only time-triggered stabilization, but also event-triggered stabilization. The approach is based on the results from [108], where robust event-triggered control strategies are derived. The focus of this approach is on networked control systems; however, it can be translated to the problem of embedded control systems.

With the development of networked control systems, e.g., swarms of AMRs or redundant and distributed flight controllers in modern aircraft, the study of systems with aperiodic sampling has emerged. Sampling times in networked systems depend on sampling jitters, packet losses, and non-determinism due to interaction between control algorithms on each controller and real-time scheduling algorithms [109]. A stability analysis on the effect of varying sampling periods in non-linear systems is presented in [110]. An approach based on fuzzy modeling is described in [111,112]. In [113], a robust stability framework for Linear Time Invariant (LTI) systems with aperiodic sampling is introduced. Research presented in [114,115] uses weakly hard real-time constraints to describe deadline misses in the stability analysis of networked control systems. The authors derived stability conditions for the given system matrices and weakly hard constraint λ. Ahrendts et al. presented a method for the verification of weakly hard real-time constraints in the context of networked systems [10]. The method extends the existing technique presented in [22] to support multiple resources (CPUs). The applicability of the presented method is demonstrated in an automotive case study. In [116], the authors propose an adaptive control method that handles overruns that occur due to delays in message transmission. The overrun handling strategy is changed during runtime depending on the delay and three different operation modes are implemented: normal mode, abort mode, and skip mode.

In recent years, the problem of control and scheduling co-design is also addressed in the context of cyber-physical systems. The main objective in system design is maximizing control performance. Research presented in [117] is focused on optimizing the control performance (minimizing the performance index) in overload conditions and it introduces an arbitration algorithm that accepts or skips jobs in order to reduce system utilization. In [33], a new task model for cyber-physical systems is introduced, which allows constraining both the system stability and control performance through sampling period and maximum tolerable number of consecutive deadline misses. Two scheduling mechanisms are introduced: offline parameter assignment and online state-aware scheduling. Offline parameter assignment determines the sampling period and weakly hard real-time constraint on the maximum number of deadline misses such that system stability is preserved. Online state-aware scheduling uses the constraints determined by the offline parameter assignment and dynamically manages deadline misses to improve the system performance. The scheduling approach is benchmarked against a similar approach presented in [118] and the rate monotonic approach. It is shown that the proposed approach outperforms the previously proposed approaches both in terms of performance and schedulability. In [15], a novel task model for cyber-physical systems is introduced that has the ability to express the stability of the underlying control system. A corresponding scheduling algorithm is designed, which guarantees the stability of the system and increases control performance. A novel approach in cyber-physical system design presented in [119] introduces an additional objective–resource efficiency. Resource efficiency is a metric that depends on the number of tasks that are schedulable on a given resource at a certain time instant. In this work, control performance is measured through the settling time. The classical real-time task model is extended in order to support two operation modes for control tasks: slow mode and fast mode. The general idea is based on the fact that longer sampling periods improve system schedulability, but this results in a worse control performance or even instability. A similar idea was described in [120]. In [119], upper and lower bounds on the acceptable period for each task, $T_{i}^{+}$ and $T_{i}^{-}$, are defined as follows:

- $T_{i}^{+}$ - period value beyond which the control system output response becomes unacceptable;
- $T_{i}^{-}$ - period value that ensures that the utilization of the task $\tau_{i}$ will not exceed the maximum allowed value.

The time instant when the operation mode for each task is switched depends on the minimum inter-arrival time of sporadic events that cause overload. The described task model is referred to as the *dual-mode* task model. The task parameters of the dual-mode model are determined by a heuristic optimization method based on a genetic algorithm. The results obtained by the dual-mode genetic algorithm are evaluated with respect to the control performance and compared to the results obtained by a traditional uniform sampling model and a dual-mode strategy with random task parameter assignment. It is shown that the dual-mode genetic algorithm increases the control performance by up to 13% in comparison with a traditional model. In [14], the authors presented a method for the verification and validation of a cyber-physical system under weakly hard real-time constraints. The applicability of the approach is demonstrated on an autonomous vehicle case study.

Table 4 summarizes the approaches for scheduling and control systems co-design reviewed in this section. The main objectives, target applications, and features are outlined.

Although they are not related to weakly hard real-time system model, there are several recent results from the area of scheduling and control co-design worth mentioning. Research presented in [121] investigates the scheduling algorithm and controller co-design for type-2 T-S fuzzy systems. A stochastic scheduling algorithm is designed through particle swarm optimization. In [122], a framework for estimation-control-scheduling co-design for wireless networked control systems is presented. Controller and scheduler optimization are performed based on a deep reinforcement learning technique. Research presented in [123] deals with time-sensitive networks. The authors propose a fixed-priority scheduling scheme and an optimization method that finds optimal sampling periods such that the settling time of the controller is minimized. In [124], a new concept is introduced, namely sub-schedulability. This concept is used for describing tasks that are not feasible but can be scheduled in a bounded time interval after their respective deadlines. Using the timing model derived from the sub-schedulability concept, the authors develop a model-predictive control method that can find a priority assignment scheme such that the performance metric is optimized while fulfilling the sub-schedulability conditions.

## 7. Conclusions

This paper provides a brief overview of the weakly hard real-time systems and the system models derived from weakly hard temporal constraints. Additionally, the techniques for performing feasibility analyses and algorithms for scheduling tasks under weakly hard real-time constraints are presented. The challenges in control and scheduling co-design are outlined, as well as the recent results in this field. There are several research problems in the context of scheduling tasks in weakly hard real-time systems: providing schedulability tests that ensure that a task set under given weakly hard constraints is schedulable, providing mechanisms for decreasing the probability of dynamic failure, designing scheduling algorithms that maximize the quality of service, etc. The previous work on these problems has emerged in two directions: the guaranteed and best-effort approaches. Further work in the context of guaranteed scheduling approaches can include improving the quality of service by employing optimization techniques in the design of heuristics for scheduling skippable jobs. The existing approaches for providing schedulability tests can be extended by considering specific task models, e.g., task sets with harmonic periods. In the context of best-effort algorithms, the possibility of employing job acceptance heuristics can be researched with the aim of minimizing the probability of dynamic failure. Additionally, most of the existing approaches for scheduling weakly hard real-time systems focus on the nm constraint only and, therefore, future research can address the implementation of scheduling algorithms that support the rest of weakly hard real-time constraints. The research gap between the area of real-time scheduling and control systems’ design is bridged by novel methods that rely on the weakly hard real-time constraints in control system stability analysis and controller design. However, there are open challenges in control and scheduling co-design. Most of the presented techniques focus on control performance optimization only, while it would be beneficial to consider additional objectives, such as resource utilization (quality of service).

## Figures and Tables

**Table 1:** Timing characteristics of the tasks in the AMR control application.

$\tau_{i}$	$T_{i}$ = $D_{i}$ (ms)	$C_{i}$ (ms)	Functionality	Constraint
$\tau_{1}$	10	2	localization	hard real-time
$\tau_{2}$	10	3	navigation	hard real-time
$\tau_{3}$	10	1	obstacle detection	hard real-time
$\tau_{4}$	20	2	battery management	weakly hard real-time: $s_{i}=5$
$\tau_{5}$	10	2	motor control	hard real-time
$\tau_{6}$	10	1	user signalization	weakly hard real-time: (1,5)
$\tau_{7}$	10	1	obstacle avoidance	hard real-time

**Table 2:** Weakly hard constraints.

	Met Deadlines	Missed Deadlines
Any order	nm	nm¯
Consecutive	nm	nm¯≡〈n〉¯

**Table 3:** A summary of the algorithms for scheduling weakly hard real-time systems.

Algorithm	Constraint	Guaranteed/Best-Effort	FP/DP	Complexity	Advantages
DBP [5]	nm	Best-effort	DP	$O(NM_{\max})$	Simple implementation
DWCS [61]	nm	Guaranteed for a special case of $D_{i}=T_{i}$ and equal loss tolerance	DP	O(N)	Considers both deadlines and loss tolerance, low computational cost
DPS [64]	nm	Guaranteed	FP	$O(NM_{\max})$	Considers general process model
GDPA [68]	nm	Guaranteed	DP	$O(N^{2})$ O(N) for GDPA-S	Maximizes the QoS
CSA [57]	nm	Guaranteed	DP	$O(NM_{\max})$ for class assignment $O(CM_{\max})$ for scheduling, *C* is the number of classes	Achieves a trade-off between QoS granularity and scalability
JCLS [16]	nm	Guaranteed	FP	O(1) if $\frac{n_{i}}{m_{i}}\geq 0.5$, otherwise exponentially depends on $m_{i}$	Used for scheduling sporadic tasks applicable to scenarios with jitter
GEBS [69]	nm	Guaranteed	FP	$O(Nm^{2}\log\left(Nm^{2}\right))$ where $m=\max\{m_{i}|\forall \tau_{i}\in T\}$	Global priority allocation scheme, takes into account emergency degrees of all tasks
BWP [47]	m−1m	Guaranteed	DP	O(N)	Simple implementation and low computational cost
RLP [72]	m−1m	Guaranteed	DP	Determined by the complexity of the EDL: $O(D_{\max}/T_{\min}\cdot N)$	Implements a mechanism for stimulating the execution of blue jobs
RLP/T [75]	m−1m	Guaranteed	DP	Schedulability test runs in $O(D_{\max}/T_{\min}\cdot N+B\left(t\right))$	Provides an acceptance test for blue jobs
BM [81]	nm, nm	Guaranteed	FP in panic mode, normal mode can use both FP and DP	Depends on the priority assignment in normal and panic modes, $O(N^{2})$ for optimal priority assignment	Considers general process model
MAA [82]	nm	Guaranteed	DP	$O(NM_{\max})$	Combines a scheduling policy that guarantees the weakly hard constraint with an arbitrary scheduling policy
MRA [83]	nm	Guaranteed	DP	O(N)	

**Table 4:** A summary of the approaches for control and scheduling co-design.

Co-Design Approach	Objective	Target Application	Features
[101]	performance index minimization	generalized control problem, expanded to linear-quadratic control problem	uses the notation of accelerable tasks that minimize performance index with every invocation
[103]	stability analysis for nonlinear control systems	applicable to a wide class of nonlinear control systems	weakly hard real-time constraints derived from sufficient condition for asymptotic stability
[106]	optimal controller design	generalized control problem	the effects of several strategies for handling deadline misses are discussed
[107]	stability analysis	generalized control problem	considers $\bar{\langle n\rangle }$ constraint and several strategies for handling deadline misses
[13]	stabilization of control systems	linear discrete-time systems	considers both time-triggered stabilization and event-triggered stabilization
[114,115]	stability analysis	networked control systems	stability conditions for arbitrary state matrices and weakly hard constraints
[117]	performance index minimization	cyber-physical systems	scheduling algorithm that skips jobs in order to reduce system utilization
[33]	optimal task parameter assignment that maximizes the worst-case control performance while guaranteeing stability	cyber-physical systems	task model that captures the relation between sampling period and weakly hard real-time constraints
[15]	bounding consecutive deadline misses and optimizing performance	cyber-physical systems	jobs are classified based on previous number of deadline misses; considers $\bar{\langle n\rangle }$ constraint
[119]	optimizing resource efficiency	cyber-physical systems	introduces an additional metric–resource efficiency and a dual-mode task model

## Data Availability

Not applicable.

## Abbreviations

The following abbreviations are used in this manuscript:

AMR	Autonomous Mobile Robot
BMS	Battery Management System
BM	Bi-Modal
BWP	Blue When Possible
CAN	Control Area Network
CPU	Central Processing Unit
CSA	Class Selection Algorithm
D-CSA	Dynamic Class Selection Algorithm
DBP	Distance-Based Priority
DMAC	Deadline-Miss-Aware Controller
DP	Dynamic Priority
DPS	Dual Priority Scheduling
DWCS	Dynamic Window-Constrained Scheduling
EDBP	Enhanced Distance Based Priority
EDF	Earliest Deadline First
EDL	Earliest Deadline as Late as Possible
FP	Fixed Priority
GDPA	Guaranteed Dynamic Priority Assignment
GDPA-S	Guaranteed Dynamic Priority Assignment-Simplified
GEBS	Global Emergency-Based Scheduling
IAE	Integral Absolute Error
IDBP	Integrated Distance-Based Priority
ISE	Integral Squared Error
ITAE	Integral Time-Weighted Absolute Error
JCLS	Job-Class-Level Scheduler
LED	Light-Emitting Diode
LTI	Linear Time Invariant
MAA	Meet Any Algorithm
MILP	Mixed-Integer Linear Programming
MRA	Meet Row Algorithm
PDS	Probability of Deadline Satisfaction
QoS	Quality of Service
RLP	Red Tasks as Late as Possible
RLP/T	Red Tasks as Late as Possible with Blue Acceptance Test
RMS	Rate Monotonic Scheduling
RTO	Red Tasks Only
S-CSA	Static Class Selection Algorithm
TBS	Total Bandwidth Server
ToF	Time of Flight
